# β-Defensin gene copy number variation in cattle

**DOI:** 10.1098/rsos.241154

**Published:** 2024-10-30

**Authors:** Ozge Sidekli, John Oketch, Sean Fair, Kieran G. Meade, Edward J. Hollox

**Affiliations:** ^1^ Department of Genetics and Genome Biology, University of Leicester, Leicester, UK; ^2^ Department of Biological Sciences, Bernal Institute, Faculty of Science and Engineering, University of Limerick, Limerick, Ireland; ^3^ School of Agriculture and Food Science, University College Dublin, Dublin, Ireland

**Keywords:** cattle, bull, β-defensins, copy number variation, fertility, *DEFB103*

## Abstract

β-Defensins are peptides with antimicrobial roles, characterized by a conserved tertiary structure. Beyond antimicrobial functions, they exhibit diverse roles in both the immune response and fertility, including involvement in sperm maturation and function. Copy number variation (CNV) of β-defensin genes is extensive across mammals, including cattle, with possible implications for reproductive traits and disease resistance. In this study, we comprehensively catalogue 55 β-defensin genes in cattle. By constructing a phylogenetic tree to identify human orthologues and lineage-specific expansions, we identify 1 : 1 human orthologues for 35 bovine β-defensins. We also discover extensive β-defensin gene CNV across breeds, with *DEFB103,* in particular, showing extensive multi-allelic CNV. By comparing β-defensin expression levels in testis from calves and adult bulls, we find that 14 β-defensins, including *DEFB103*, increase in expression during sexual maturation. Analysis of β-defensin gene expression levels in the caput of adult bull epididymis, and β-defensin gene copy number, in 94 matched samples shows expression levels of four β-defensins are correlated with genomic copy numbers, including *DEFB103*. We therefore demonstrate extensive CNV in bovine β-defensin genes, in particular *DEFB103*, with potential functional consequences for fertility.

## 1. Introduction

One of the largest sub-classes of the defensin protein family, β-defensins were first characterized as short cationic peptides produced by many multi-cellular organisms with an antimicrobial role [1,2]. β-Defensins peptide sequences are very diverse, with the conservation of a core glycine and aspartic acid residue and six cysteines, which form three disulphide bridges in the mature protein leading to a conserved tertiary structure characteristic of all β-defensins [3]. Secreted at epithelial and mucosal surfaces, it has become increasingly clear that defensins in fact have adopted a multitude of additional roles over the course of their evolution. Evidence now shows that defensins interact with other components of the innate and adaptive immune system, in often complex and contradictory ways [2]. For example, human β-defensin 3 (hBD-3) has dual roles in modulating the immune response. It can directly act as a chemoattractant for monocytes by binding to the CCR2 receptor, similar to traditional chemokines [4,5]. Additionally, hBD-3 can indirectly promote a pro-inflammatory response by stimulating the production of various cytokines and chemokines from other cells, thereby amplifying the recruitment and activation of immune cells [6].

β-Defensins have roles beyond modulation of the immune response. It is clear that, in humans at least, some β-defensins are expressed in different regions of the epididymis post-puberty, with many only being expressed in the epididymis [7,8]. Empirical evidence has implicated specific β-defensins in sperm maturation, function and ultimately regulating male fertility [9,10]. The β-defensin best characterized in its role in male fertility is β-defensin 126 (*DEFB126*), which has been extensively characterized in humans and macaques [11]. *DEFB126* protein is secreted by the epididymis, is highly glycosylated and subsequently adsorbed onto the surface of sperm as they travel through the epididymis. Through the addition of a negative charge, *DEFB126* facilitates penetration of cervical mucus by the sperm and possibly protects the sperm from female immune recognition [12]. The adsorbed *DEFB126* is subsequently shed during capacitation to allow fertilization to occur [13,14]. *DEFB126* has also been shown to affect sperm motility in cattle via the enhancement of sperm binding to oviductal epithelium [15] and the prevention of sperm agglutination [16]. Other β-defensins have been shown to affect fertility, and given their widespread expression in the male reproductive tract, much remains to be discovered about the functional role of these proteins [17,18]. Beyond *DEFB126*, most knowledge so far is from the analysis of β-defensins and fertility in rodents. A mouse knockout of a cluster of nine β-defensins in mice renders male mice infertile [10], with evidence showing specific sperm defects such as reduced motility, increased fragility, premature capacitation, increased spontaneous acrosome reaction and reduced ability to bind the zona pellucida.

Across mammals, different members of the β-defensin gene family show extensive copy number variation (CNV) between individuals [1]. This has been best characterized in humans [19,20], but CNV has been characterized in detail in macaque [21] and has been detected in genome-wide scans in dogs [22], pigs [23], chickens [24] and cattle [25]. CNV of the β-defensin gene *DEFB4* has phenotypic consequences in humans, as it has been shown to be associated with its encoded protein levels and mucosal antimicrobial activity in the cervix of the CNV [26], and associated with the risk of the inflammatory skin disease psoriasis [27]. However, a robust association of β-defensin CNV with a phenotype outside humans has not yet been shown, nor has an association been shown with a phenotype reflecting the reproductive role of β-defensins rather than their antimicrobial or inflammatory roles.

In this study, we have focused on characterizing the CNV of β-defensins in domestic cattle (*Bos taurus*). For the dairy industry, in particular, a focus on improving food security through higher milk yields has led to a significant decline in fertility [28]. Fertility is a critical trait for sustainable animal production systems and significant variation in bull fertility can account for low pregnancy rates of individual bulls, and also, although multiple single nucleotide polymorphisms (SNPs) have been associated with fertility, the ability to predict poor bull fertility remains elusive despite the extensive use of sperm motility and morphology quality control checks prior to the release of semen into the field [29,30]. Together with the emergence of artificial insemination activities, this has renewed the interest and importance of understanding the role of genetic variation in reproductive traits, including bull subfertility. Therefore, understanding the extensive genetic variation of the β-defensin family, extremely important in mammalian reproduction, in cattle is an important step in understanding its phenotypic consequences for reproduction.

In cattle, 57 β-defensins have been previously identified based on sequence similarity, including the genes encoding tracheal antimicrobial peptide (*TAP*) and lingual antimicrobial peptide (*LAP*) [3]. These β-defensin genes occur in four clusters on chromosome 8 (cluster A), chromosome 13 (cluster B), chromosome 23 (cluster C) and chromosome 27 (cluster D), orthologous to the human β-defensin clusters on chromosome 8 (proximal), chromosome 20, chromosome 6 and chromosome 8 (distal) [31]. By mapping whole-genome short-read sequencing to a cattle genome assembly, 11 β-defensins were reported to show CNV, with average copy numbers suggesting extensive gene duplication [25].

The development of cattle genomic resources and improvement of the cattle reference genome have important consequences for the accuracy of CNV inference. Bickhart *et al.* [25] used Btau_4.0 as the reference genome, and the subsequent genome assembly (UMD3.1) allowed the incorporation of previously unplaced genomic contigs. The most recent assembly (ARS-UCD1.2), which incorporates long sequencing reads from Pacific Biosystems technology, represents a 200-fold improvement in sequence contiguity and a 10-fold improvement in sequence accuracy per base, compared with UMD3.1 [32]. This improved contiguity will have particularly improved regions with segmental duplications prone to CNV, which are known to be challenging to assembly using just short-read sequences. Whole-genome short-read sequencing data provided by the 1000 Bulls consortium has allowed an assessment of the genome-wide sequence variation between and within cattle breeds [33]. Short-read sequencing data can also be used to determine the nature and extent of CNV in a genome. Taken together, these genomic resources provide an ideal starting point for a focused analysis on the CNV of β-defensins in cattle.

In this work, we comprehensively catalogued the β-defensin genes in the latest high-contiguity bovine genome assembly and identified human orthologues and recent β-defensin expansions in the bovine lineage by constructing a phylogeny.

We identify the range and extent of CNV in β-defensin genes across seven different (Holstein, Hereford, Charolais, Limousin, Simmental, Angus, Swiss Braunvieh and their cross-breeds) breeds, focusing particularly on Holsteins, the primary dairy breed worldwide. We develop digital droplet polymerase chain reaction (ddPCR) assays to extend this analysis to unsequenced bulls and measure copy numbers of Holstein and Jersey bulls in the Irish dairy herd. We then analyse the expression levels of β-defensin genes in the caput epididymis and the relationship of CNV with gene expression.

## 2. Methods

### 2.1. 1000 Bulls samples

Whole-genome sequences (WGS) were downloaded from 1000 Bulls Consortium data at the European Nucleotide Archive (ENA, https://www.ebi.ac.uk/ena), focusing on Holstein–Friesian (*n* = 30), but also including Hereford (*n* = 6), Charolais (*n* = 26), Limousin (*n* = 19), Simmental (*n* = 9) and Angus (*n* = 10) breeds [34] (electronic supplementary material, table S1).

### 2.2. Other data sources

RNA-seq data for Holstein (adult, *n* = 24) and Hereford (calf, *n* = 3; adult, *n* = 7) were obtained from ENA under accession numbers PRJNA566324, PRJNA760322, PRJNA263600, PRJNA379574 and PRJNA526257 (electronic supplementary material, table S2). Data from the Swiss Braunvieh and their cross-breeds (*n* = 103), used for correlation analysis of CNV with gene expression at the transcript level in caput epididymis, were generated by Mapel *et al.* [35] (electronic supplementary material, table S3). From this dataset, a total of nine samples were removed from the study, seven due to insufficient coverage in the DNA sequence and two due to corrupt raw files or missing second reads in the RNA sequence. In total, 94 samples were retained for analysis, encompassing both transcript-level gene expression and CNV assessment. These datasets are accessible on the ENA under the accession number PRJEB46995 (electronic supplementary material, table S3).

### 2.3. DNA samples and genomic DNA extraction

Genomic DNA was extracted using a standard phenol–chloroform method from semen from Irish Holstein–Friesian bulls (*n* = 20) and Jersey bulls (*n* = 15) and positive control bulls (*n* = 4) selected from WGS analysis. The genomic DNA concentration was determined by measuring the fluorescence signal using the Qubit 2.0 Fluorometer.

### 2.4. β-Defensin search and alignment

Using keywords ‘Human’, ‘Bovine’ and ‘β-defensin; DEFB’, β-defensin gene and β-defensin predicted protein sequences were retrieved from Ensembl [36], National Center for Biotechnology Information (NCBI; [37]), UniProt (https://www.uniprot.org/; [38] and University of California Santa Cruz (UCSC) Genome Browser (https://genome.ucsc.edu/; [39]) by querying the reference genomes ARS-UCD1.2 for cattle and GRCh38 for human.

A total of 66 bovine and 39 human β-defensin gene sequences and predicted protein sequences were identified. To refine the dataset, the signal peptides of the protein sequences were cleaved using resources from the UniProt website, and identical sequences, those that did not map to the latest reference genome, and those lacking a predicted start codon, were excluded from the analysis. A curated dataset consisting of 55 β-defensin predicted protein sequences in cattle (electronic supplementary material, table S4) and 37 β-defensin predicted protein sequences in humans (table 1) were aligned using Clustal Omega [40].

**Table 1.** Human orthologues of bovine β-defensin genes.

Ensembl gene ID	symbol	location ARS-UCD1.2 assembly	protein	protein accession	orthologues
not available	*DEFB43*	chr8:7,449,722–7,456,717	BBD43	XP_024852154.1	HBD131A
ENSBTAG00000054255	*DEFB134*	chr8:7,469,680–7,473,928	BBD134	XP_024852155.1	HBD134
ENSBTAG00000051632	*DEFB136*	chr8:7,499,869–7,500,896	BBD136	XP_024852156.1	HBD136
ENSBTAG00000049760	*DEFB132−1*	chr13:60,755,471–60,757,652	BBD132-1	XP_005215029.4	HBD132
ENSBTAG00000048288.2	*DEFB129−2*	chr13:60,772,966–60,775,142	BBD129-2	ENSBTAT00000064705.2	HBD129
ENSBTAG00000051173	*DEFB128*	chr13:60,785,898–60,787,480	BBD128	ENSBTAT00000068291.1	HBD128
ENSBTAG00000054958	*DEFB127*	chr13:60,793,742–60,796,520	BBD127	XP_005215144.1	HBD127
ENSBTAG00000051033	*DEFB126*	chr13:60,806,515–60,811,042	BBD126	ENSBTAT00000071785.1	HBD126
not available	*DEFB125A*	chr13:60,828,555–60,835,043	BBD125A	XP_024856951.1	HBD125
ENSBTAG00000053093	*DEFB125*	chr13:60,828,987–60,829,208	BBD125	G8CY10	HBD125
ENSBTAG00000049491	*DEFB115*	chr13:60,873,647–60,884,399	BBD115	XP_024857141.1	HBD115
ENSBTAG00000050556	*DEFB29*	chr13:60,893,940–60,905,009	BBD29	XP_002692327.1	none
ENSBTAG00000052478	*DEFB116*	chr13:60,920,317–60,925,563	BBD116	XP_010809939.1	HBD116
ENSBTAG00000048009	*DEFB117*	chr13:60,956,394–60,959,265	BBD117	ENSBTAT00000063334.2	HBD118
ENSBTAG00000052743	*DEFB118*	chr13:60,979,467–60,979,700	BBD118	G8CY17	HBD118
not available	*DEFB119−1*	chr13:60,981,163–60,990,988	BBD119-1	NP_001095131.1	HBD119
ENSBTAG00000003364	*DEFB119−2*	chr13:60,989,366–60,990,988	BBD119-2	NP_001095829	none
ENSBTAG00000048949	*DEFB121*	chr13:61,007,870–61,009,276	BBD121	XP_005215031.1	HBD121
ENSBTAG00000027384	*DEFB122A*	chr13:61,019,572–61,023,625	BBD122A	NP_001095809.1	none
ENSBTAG00000027383	*DEFB122*	chr13:61,030,365–61,034,955	BBD122	NP_001071575.1	none
not available	*DEFB123−2*	chr13:61,038,268–61,052,092	BBD123-2	XP_024856654.1	HBD123
ENSBTAG00000020555	*DEFB123−1*	chr13:61,041,375–61,051,806	BBD123-1	NP_001095813.1	HBD123
ENSBTAG00000031254	*DEFB124*	chr13:61,067,234–61,069,998	BBD124	NP_001095830.1	HBD124
ENSBTAG00000054219	*DEFB114*	chr23:22,612,721–22,615,376	BBD114	XP_024839622.1	HBD114
ENSBTAG00000054396	*DEFB113*	chr23:22,632,551–22,635,067	BBD113	XP_024839712.1	HBD113
not available	*DEFB110−1*	chr23:22,644,099–22,656,635	BBD110-1	XP_024839746.1	HBD110
ENSBTAG00000051453	*DEFB110−2*	chr23:22,651,406–22,656,635	BBD110-2	XP_002697354.1	none
ENSBTAG00000046711	*DEFB112*	chr23:22,662,983–22,671,406	BBD112	XP_024839747.1	HBD112
ENSBTAG00000050611	*DEFB1−2*	chr27:5,960,716–5,968,525	BBD1-2	ENSBTAT00000068469.1	none
ENSBTAG00000048171	*TAP*	chr27:6,013,830–6,015,648	TAP	NP_777201.1	none
ENSBTAG00000052579	*DEFB103B*	chr27:6,023,993–6,025,303	BBD103B	XP_010818521.1	HBD103B/HBD-like
ENSBTAG00000034952	*SPAG11B*	chr27:6,045,534–6,050,941	BSPAG11B	XP_005225990.1	H-SPAG11B
ENSBTAG00000048847	*DEFB104*	chr27:6,069,928–6,074,858	BBD104	ENSBTAT00000075330.1	HBD104A
ENSBTAG00000049070	*DEFB106A*	chr27:6,076,779–6,079,838	BBD106	ENSBTAT00000075644.1	HBD106B-2
not available	*DEFB15*	chr27:6,076,989–6,079,896	BBD15	XP_024842096.1	HBD106B-2
ENSBTAG00000052911	*DEFB105*	chr27:6,082,209–6,084,799	BBD105	XP_015316308.1	HBD105A
ENSBTAG00000054607	*DEFB107A*	chr27:6,090,910–6,095,691	BBD107A	XP_002698669.1	HBD107A/HBD106B-1
ENSBTAG00000053557	*DEFB4A*	chr27:7,138,963–7,140,882	BBD4A	NP_777200	none
not available	*DEFB5*	chr27:7,139,081–7,140,848	BBD5	NM_001130761.1	none
ENSBTAG00000033545	*EBD*	chr27:7,165,176–7,180,422	EBD	NP_783634	none
ENSBTAG00000051223	*DEFB130−1*	chr27:6,192,946–6,196,744	BBD130	XP_024842219.1	HBD130A
ENSBTAG00000053889	*LAP*	chr27:6,234,006–6,235,870	LAP	NP_982259.3	none
ENSBTAG00000046937	*DEFB33*	chr27:6,238,997–6,242,941	BBD33	NP_001161717	none
ENSBTAG00000050630	*DEFB13*	chr27:6,326,696–6,328,616	BBD13	NP_001311470.1	none
ENSBTAG00000050419	*DEFB103A*	chr27:6,432,334–6,433,571	BBD103A	XP_015316312.1	HBD103B/HBD-like
ENSBTAG00000048737	*DEFB10*	chr27:6,596,623–6,598,299	BBD10	NP_001108556.1	none
not available	*DEFB3*	chr27:6,676,309–6,723,692	BBD3	NP_001269510	none
ENSBTAG00000051383	*DEFB7*	chr27:6,676,383–6,678,273	BBD7	NP_001095832.1	none
not available	*DEFB6*	chr27:6,676,387–6,996,057	BBD6	NP_001300920	none
not available	*DEFB9*	chr27:6,676,540–6,678,161	BBD9	P46167	none
ENSBTAG00000052312	*DEFB1−1*	chr27:6,721,772–6,723,692	BBD1-1	NP_001311473.1	HBD1
ENSBTAG00000053456	*DEFB402*	chr27:6,855,327–6,856,946	BBD402	Q5W5I5	none
ENSBTAG00000054169	*DEFB108B*	chr27:7,272,455–7,275,948	BBD108B	XP_024842173.1	HBD108B
not available	*DEFB109*	chr27:7,281,838–7,289,252	BBD109	XP_024842216.1	HBD109A

### 2.5. Phylogenetic analysis

The multiple protein sequence alignment of bovine (*n* = 54; BBD129-1 was omitted due to the same mature protein sequence as BBD129-2) and human β-defensins (*n* = 37) was used to construct a phylogenetic tree by maximum-likelihood using IQ-TREE v. 1.6.12 [41], using the JTTDCMut+I+G4 model (revised Jones–Taylor–Thornton matrix, allowing for invariable sites and a discrete four-state gamma model for site rate heterogeneity), and 1000 bootstrap replicates.

### 2.6. Copy number analysis using whole-genome short-read sequences

Fastq reads were aligned to the reference genome ARS-UCD1.2 using BWA-mem2 v. 2.2.1 [42]. Alignment quality was assessed using QualiMap, v. 2.3 [43]. Mapped genomes were sorted using SAMtools (v. 1.17) to generate BAM files. PCR duplicates were removed using Picard v. 2.6.0 (https://broadinstitute.github.io/picard/). Three samples (SRR1262661, SRR1262663 and SRR1262668) were excluded as their mean coverage fell below a threshold of 10×.

Genomic locations of the 55 β-defensin genes analysed were converted into a .bed file, and a custom Perl script counted mapped sequence reads for the regions defined in the .bed file. Read counts were normalized against a reference gene (*TP53*), then normalized by dividing by the average number of reads for each gene and then multiplied by two to get an estimate of copy number per diploid genome. Threshold values of less than 1.5 and greater than 2.5 were used to identify deletions and duplications, respectively, based on the expected copy number of two for the normal diploid genome. Three copies of the *DEFB103* gene are assembled in ARS-UCD1.2 (*DEFB103A*, *DEFB103A*-like and *DEFB103B*), so an expected copy number of six was used for the normal diploid genome.

### 2.7. Copy number analysis using digital droplet polymerase chain reaction

A subset of 31 β-defensin genes with previous evidence suggesting a link with fertility were selected (electronic supplementary material, table S5), including the multi-copy *DEFB103*, with a single primer pair designed to bind to all three annotated *DEFB103* genes. PCR primers were designed to amplify products between 160–280 base pairs for 31 selected β-defensin genes and 80 base pairs for the reference gene *TP53*. Primers were validated using the *in silico* PCR tool in the UCSC Genome Browser to ensure that they were unique to the target sequence and that common sequence variants were absent (electronic supplementary material, table S5).

ddPCR was performed using the EvaGreen Supermix QX200™ Droplet Digital PCR (Bio-Rad Laboratories) according to the manufacturer’s instructions, with QuantaSoftTM v. 1.7 and R v. 4.1.0, used to calculate the normalized copy number. In this assay, excluding *DEFB103*, a normal diploid CN of two was used as a reference, and thresholds of less than 1.5 and greater than 2.5 were used to identify deletions and duplications, respectively. A diploid copy number of six was used as the reference for *DEFB103*, and distinct threshold values of less than 4.5 and greater than 7.5 were employed to identify the low copy number and high copy number, respectively, of this gene.

## 3. Results

### 3.1. Genomic organization of β-defensin genes in cattle

Our first aim was to infer the evolution of bovine β-defensin genes and identify the closest human orthologue, if possible. We identified 55 bovine-predicted annotated β-defensin proteins and 37 predicted annotated human β-defensin proteins. Genes encoding the bovine proteins were annotated to one position on the ARS-UCD1.2 genome assembly, except BBD103, which was annotated three times to three separate regions. Aligning the predicted proteins (BBD103A, BBD103A-like and BBD103B) shows that BBD103B is most similar to human β-defensin 103, with BBD103A encoding an extra 14-amino acid region at the N-terminus, likely to disrupt effective processing and secretion of any protein, and BBD103A-like lacking an initial methionine. For phylogenetic analysis, we used the two predicted full-length proteins.

**Figure 1.**
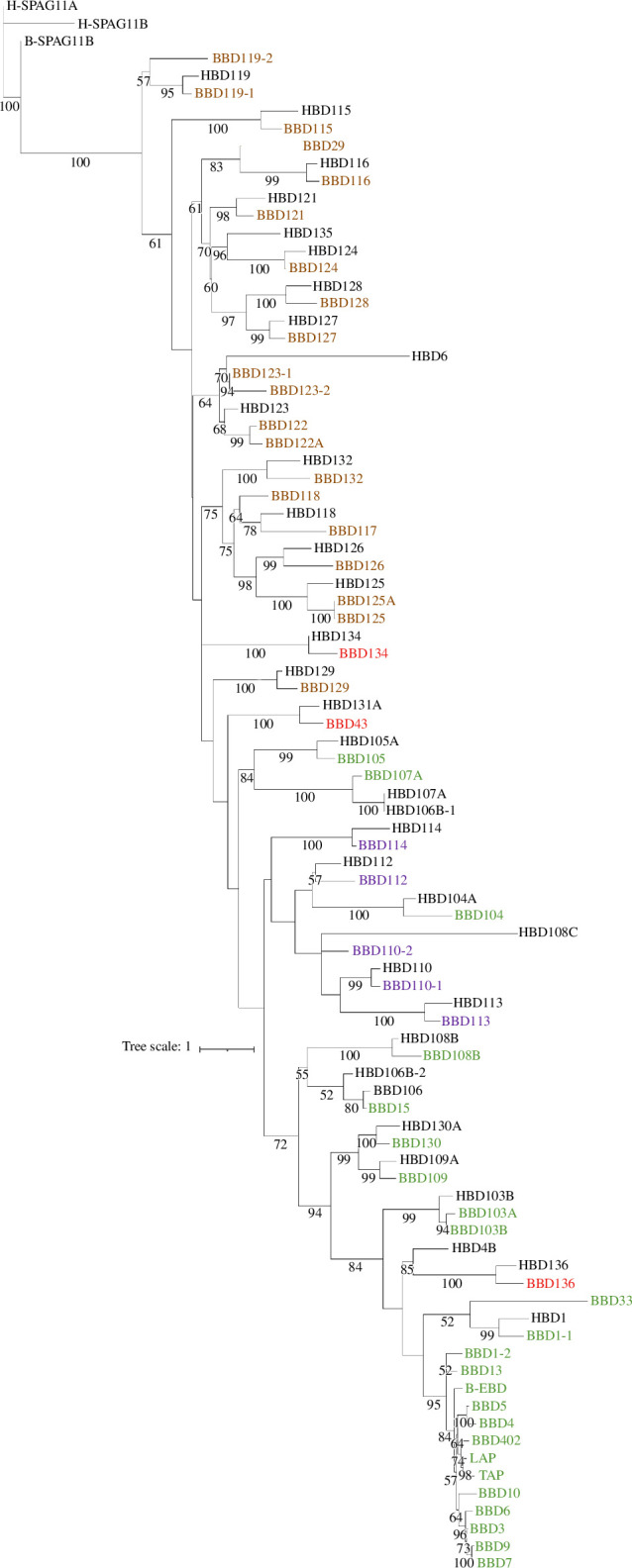
Phylogenetic tree of human and bovine β-defensin proteins. Bovine protein names are coloured according to the chromosomal location of their genes (brown: chromosome 13, green: chromosome 27, red: chromosome 8, purple: chromosome 23). Tree scale is indicated.

An unrooted maximum-likelihood phylogenetic tree from predicted protein sequences shows that the bovine β-defensins broadly cluster in the tree according to their genomic clusters, indicating a pattern of gene duplication and divergence at those clusters (figure 1). There are three exceptions—BBD134, BBD104 and BBD136, whose positions on the phylogenetic tree are incongruous compared with their cluster position of their encoding genes in the genome. The bootstrap values supporting their position in the tree are high (mostly over 70%), suggesting that this is not an error in the tree, and given the high quality of the reference genome, this points to an ancestral rearrangement after duplication and divergence of the β-defensin clusters.

The phylogenetic tree also shows that 35 bovine β-defensins have a human orthologue (table 1), with a cluster of 13 genes on chromosome 27 (cluster D), including the *TAP* and *LAP* having no one-to-one human orthologues and being closely related to each other, suggesting duplication and divergence of this group after the divergence of primates and artiodactyls. The orthologues of the three bovine β-defensins that show incongruity between tree position and genomic cluster position of their gene (BBD134, BBD104 and BBD136) have human orthologues whose genes are also incongruent with their genomic cluster, suggesting that any evolutionary rearrangement occurred prior to primate-artiodactyl divergence.

### 3.2. Analysis of copy number variation of β-defensins in cattle

To understand the CNV of β-defensin genes in cattle, we used a gene-focused approach to determine gene copy numbers from short-read sequencing alignments for 191 cattle across seven breeds. We found evidence of CNV for all the genes analysed (electronic supplementary material, figure S1), with diploid copy numbers usually ranging from one (heterozygote deletion) to three (heterozygote duplication; electronic supplementary material, table S2). There are exceptions—for example, *DEFB103* is extensively multi-allelic and shows a high copy number range of 1–29 (electronic supplementary material, figure S2), and *DEFB402* ranges from complete absence to five copies. This emphasizes that, in most breeds, deletions and duplications of the genes are relatively uncommon, such that homozygous individuals are unusual. For most genes, some breeds did not show any copy number change—for example, in Angus 34 β-defensins did not show CNV, although this is in a relatively small sample set of 10 bulls.

In contrast, the Holstein breed (*n* = 27) and Swiss Braunvieh breed and their crosses (*n* = 94) showed CNV of all β-defensins, suggesting that the sample size of other breeds has limited the ability to detect rarer copy number variants.

In order to investigate whether any genes were on a copy number variable region together, we tested the degree to which CNV across all pairs of genes covaried in the WGS dataset. The rationale for this is that if two genes are on the same copy number variable region, then, across the 191 cattle, loss or gain of sequence read depth will be strongly correlated. By calculating the correlation coefficient between every pair of genes and using a threshold of 0.9, we found three pairs of genes to covary: *DEFB7* and *DEFB9* (*r* = 1.00), *DEFB15* and *DEFB106A* (*r* = 0.99) and, unsurprisingly because they are alternative transcripts from the same genomic region, *DEFB110.1* and *DEFB110.2* (*r* = 0.97). *DEFB7* and *DEFB9* are similar genes from the recently expanded cluster D on chromosome 27, and their genomic locations overlap (table 1). *DEFB15* and *DEFB106A* also map to cluster D and also overlap (table 1). Using a lower threshold of *r* = 0.85 identifies *DEFB113, DEFB112* to *DEFB110* as a possible copy number variable block on cluster C. However, full resolution of breakpoints of these complex and highly variable regions will require long-read sequencing and multiple complete genome assemblies.

We analysed β-defensin CNV in Holstein and Jersey breed cattle from the national Irish dairy herd by developing a ddPCR for a subset of β-defensin genes, selected for their likely importance in fertility. After validating assay accuracy using matched WGS data from the same individual (electronic supplementary material, figure S3), and validating assay precision using repeat testing, we typed 20 Holstein bulls and 15 Jersey bulls (electronic supplementary material, figure S4). We confirmed CNV in these cattle, including the extensive multi-allelic CNV of *DEFB103*. This shows that β-defensin genes are extensively copy number variables in the Irish national dairy herd and may be responsible for phenotypic variation across individuals.

### 3.3. Expression of β-defensins in testis from calf and adult

Given previous evidence that most β-defensins are expressed in the testis, we wished to examine this in cattle in detail across our catalogue of β-defensins. We decided to use published RNA-seq data from three (13-week-old) calves and seven adult bulls (3 and 9 years and 11 months old) to perform a focused analysis of β-defensin expression in the testis during sexual maturation. By quantifying transcript levels, expression of 27 β-defensin genes was detected out of 44 (electronic supplementary material, table S6), with 14 showing a higher expression (false discovery rate (FDR) less than 0.1) in adult testis compared with calf testis in Hereford bulls (table 2).

**Table 2.** β-Defensin genes upregulated in adult bull testes.

gene	gene ID	log fold change	false discovery rate
*DEFB122A*	ENSBTAG00000027384	12	1.05 × 10^–17^
*DEFB33*	ENSBTAG00000046937	11.75	5.51 × 10^–14^
*DEFB122*	ENSBTAG00000027383	11.42	1.16 × 10^–12^
*DEFB119*	ENSBTAG00000003364	9.97	3.84 × 10^–19^
*DEFB123*	ENSBTAG00000020555	7.23	8.53 × 10^–6^
*EBD*	ENSBTAG00000033545	6.42	1.36 × 10^–6^
*SPAG11B*	ENSBTAG00000034952	6.33	9.40 × 10^–8^
*LAP*	ENSBTAG00000053889	5.92	2.50 × 10^–5^
*DEFB106A*	ENSBTAG00000049070	5.62	1.84 × 10^–4^
*DEFB7*	ENSBTAG00000051383	4.88	3.4 × 10^–3^
*DEFB104*	ENSBTAG00000048847	4.04	2.97 × 10^–4^
*DEFB29*	ENSBTAG00000050556	3.98	0.01
*DEFB103B*	ENSBTAG00000052579	3.76	0.01
*DEFB112*	ENSBTAG00000046711	2.57	4.39 × 10^–3^

Previous work has shown that some β-defensins, including those involved in fertility, show an extended C-terminal tail with the potential to be heavily glycosylated and to form part of the sperm glycocalyx, for example, β-defensin 126 [44]. We analysed the 14 β-defensin predicted protein sequences whose genes show an increase in expression at sexual maturity for the presence of an extended C-terminal tail and glycosylation potential (electronic supplementary material, table S7). Of the 14, two (BBD119 and SPAG11B) showed long C-terminal tails with at least one potential O-glycosylation site, four (BBD122, BBD122A, BBD104 and BBD29) showed a long-terminal tail with no glycosylation sites and eight (BBD123, BBD112, BBD33, BBD15, BBD7, BBD103, EBD and LAP) showed no long C-terminal tail. Notably, SPAG11B (Bin1b), which also has a long C-terminal tail and potential glycosylation sites, was associated with sperm motility (as reviewed by Dorin & Barratt [17]). The diversity of predicted structures of the upregulated β-defensins suggests that they have a variety of roles in the testis, potentially including glycocalyx formation and direct antimicrobial action.

### 3.4. Expression of β-defensin in caput region of epididymis

**Figure 2.**
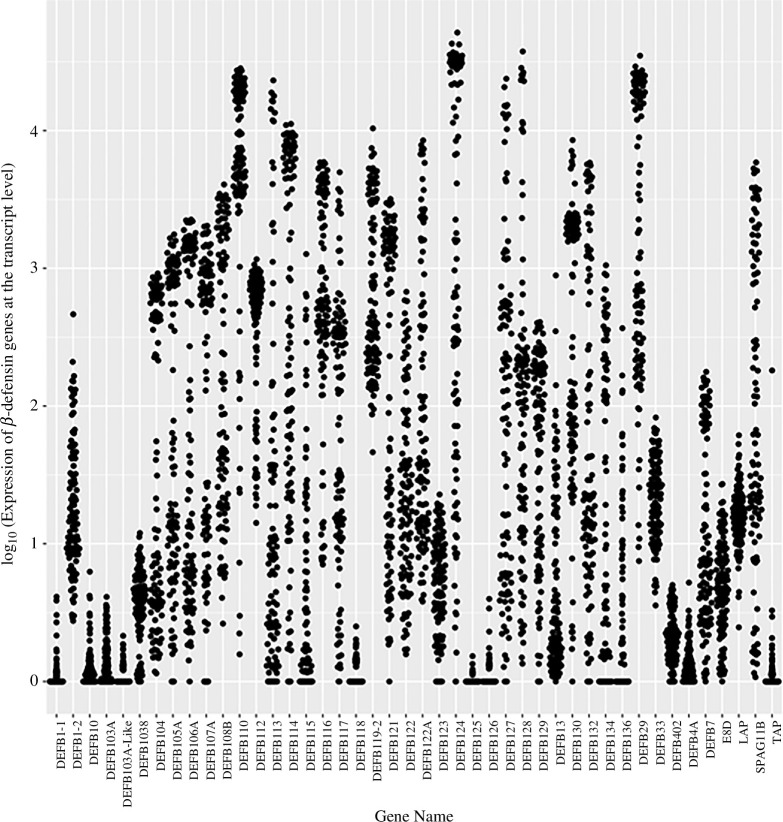
Expression of bovine β-defensin genes in the caput of the epididymis.

The epididymis is a complex organ, and any expression data based on nucleic extraction from the whole organ will not be able to distinguish expression in different cell types, which can limit interpretation of expression levels of particular genes. For example, previous work has shown that β-defensins are expressed differently in different mouse testis cell types and segments of the epididymis [45]. Additionally, β-defensin mutation in rodent sperm from the cauda epididymis has been linked to female infertility [46]. To address this in part, we used RNA-seq data from the caput of the epididymis of 94 bulls to assess the transcript levels of 44 β-defensin genes (figure 2). All 44 β-defensins are expressed to a greater or lesser extent in the caput, with, for example, *DEFB124* and *DEFB29* expressed at high levels and *TAP* and *DEFB118* expressed at low levels. Indeed, a previous analysis of this data found that four β-defensins (*DEFB110, DEFB124, DEFB121* and *DEFB114*) were among the 50 most tissue-specific/tissue-enriched transcripts in the epididymis [35]. However, all expressed defensins show a high degree of variation between different bulls for any particular β-defensin, often over three orders of magnitude. For some genes, such as *DEFB134* and *DEFB402*, this range includes several bulls who show no expression of the gene at all.

It is likely that the pattern of β-defensin gene expression changes along the epididymis from caput to cauda. Strong regional expression of β-defensins in the epididymis has been shown in cattle [47], rams [48] and rats [49,50]. *DEFB103* is strongly expressed in the cauda of cattle epididymis (as is its orthologue *Defb14* in rats), as shown by reverse transcription (RT)-PCR [31], but our analysis shows that it is weakly expressed in the bovine caput. Therefore, absence or low expression of a particular gene in the caput does not reflect on expression levels elsewhere in the epididymis, nor on its potential role in fertility. Nevertheless, the extensive variation of expression of the defensins could potentially have consequences on fertility and reproduction-associated phenotypes.

### 3.5. Correlation of gene expression with gene copy number

The individual variation in expression level will be due to a mixture of genetic, environmental and possibly technical factors. Given the extensive CNV of these genes, this is a strong candidate to explain at least part of any genetic variation contributing to the variation in expression levels between individuals. This could be through a gene dosage effect, where an increase in the number of copies of a gene leads to a concomitant increase in transcript level, or through more complex effects such as disrupting expression levels through higher-order chromatin structure at the locus.

**Figure 3.**
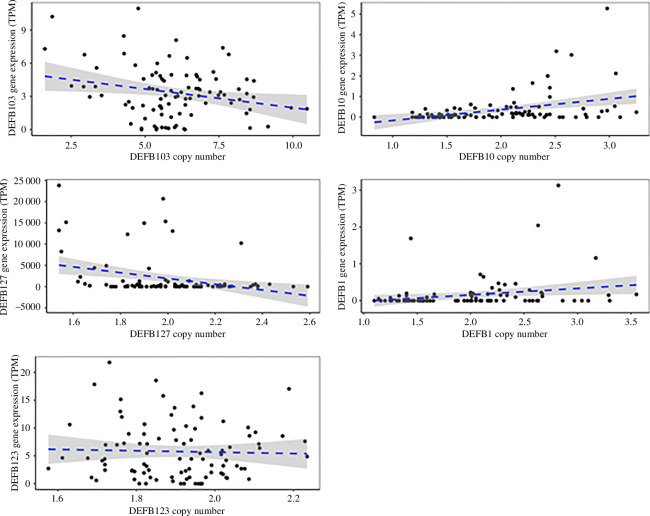
CNV and gene expression of selected β-defensins. *DEFB10*, *DEFB127*, *DEFB1-1*, *DEFB103* and *DEFB123* genes shown. The *X*-axis represents the diploid copy number, while the *Y*-axis indicates the expression of the gene in transcripts per million (TPM). *DEFB123* is not copy number variable (all copy number values are approx. 2) and shows an example of the range of expression values of a non-CNV gene.

Correlating the expression levels of the 44 genes against estimates of gene CNV identified four genes (*DEFB103, DEFB10, DEFB127* and *DEFB1*) with a nominal *p* value less than 0.05 where expression was correlated with gene copy number (electronic supplementary material, table S7 and figure 3). For *DEFB1-1*, examination of the data shows that the correlation is driven by outlying higher expression levels in an overall very low-expressed gene. However, for the remaining three genes, *DEFB10* shows a positively correlation but two (*DEFB127* and *DEFB103*) show a negative correlation. The positive correlation can be explained at least in part by a gene dosage effect, and given that the protein encoded by *DEFB10*, BBD10, is part of the recently duplicated clade including LAP and TAP, is upregulated by vitamin D and is likely to be antimicrobial [51], although this has not been shown directly. *DEFB103* orthologues have been shown to be antimicrobial but also involved in cell signalling [52]. *DEFB127* has a long C-terminal tail (45 amino acids after last cysteine) and four predicted glycosylation sites. Given the evidence suggesting that β-defensins bind to sperm via glycosylation in the long C-terminal region [12,44,53], DEFB127 may potentially play a role in fertility through interactions with sperm.

## 4. Discussion

In this study, we have fully characterized the complement of β-defensin genes in cattle and have constructed a phylogenetic tree to identify their human orthologues and β-defensin family expansions in the bovine lineage. This builds on previous work, starting with the identification of the genes, including *LAP* and *TAP*, that we know represent a recent gene family expansion on chromosome 27 (part of cluster D) [54]. These were shown initially to be expressed in udders. A more complete search of the cattle genome assembly UMD 3.1 identified 57 β-defensin open reading frames, of which 53 were full-length [3]. In this study, by using the recent high-quality ARS-UCD1.2 genome assembly, we refine this complement to 55 full-length β-defensin genes and identify 1 : 1 human orthologues for 35 bovine β-defensin genes, suggesting conservation of function of these β-defensins for approximately 90 million years. In contrast, cluster D of β-defensins on chromosome 27 has evolved by more recent duplication and divergence. This chromosome 27 β-defensin cluster D generally has a broader expression profile across tissues compared with older β-defensins, which are generally restricted, or most highly expressed, in the testis [3]. Cluster D includes *LAP* and *TAP*, encoding for lingual antimicrobial peptide and tracheal antimicrobial peptide respectively, and these two proteins have been shown to be directly antimicrobial and involved in the immune response to various diseases. Orthologues of at least some of these genes have been identified in sheep [55,56], goat and water buffalo (as reviewed by Meade *et al*. [57]).

We also show that genes encoding three different predicted BBD103 proteins map to three different regions on the ARS-UCD1.2 genome assembly. BBD103B is short and most similar to human β-defensin 103, suggesting that it is its orthologue. BBD103A has a 14-amino acid segment at the N-terminus, which is likely to disrupt any signal sequencing processing and consequent secretion, and a long C-terminal tail. Compared with BBD103B, BBD103-like does not have a predicted initial methionine. It is likely that only BBD103B is a functional protein; however, all three transcripts are detected in the caput of the epididymis, and the CNV we observe for *DEFB103* does not distinguish between the three related genes. Multiple copies of the *DEFB103* gene are likely to encode a mixture of the three related transcripts: A, A-like and B, but analysing the diversity within the CNV requires further investigation. Our observation of *DEFB103* CNV might explain in part the observations in previous publications on this gene—Mirabzadeh-Ardakani *et al.* [58] analyse *DEFB103B*, but document extra copies of *DEFB103* in earlier genome assemblies [58].

The genes on clustered bovine chromosome 8*—DEFB136*, *DEFB134* and *DEFB43* (also known as *DEFB131*)—(cluster A) do not form an evolutionary cluster but share apparent evolutionary relationships with genes on other chromosomes. They preserve synteny, but in inverted order, with their orthologues in the sheep genome [55], synteny with their orthologues in dogs [31] and are in a highly structurally variable region in humans. A full analysis of β-defensin evolution in artiodactyls will clarify the evolution of cluster A and the chromosome 27 cluster D.

Previous work has suggested that many β-defensins show CNV across cattle breeds [25]. However, although the existence of CNV was extensively validated, estimates of copy number were high, on five cattle with low sequencing coverage, shorter read lengths and were based on an early cattle genome assembly. We took a gene-focused approach to investigate CNV in 191 cattle across seven breeds from the 1000 Bulls project and previously published data and confirmed/extended our analysis into the Irish national herd of Holstein and Jersey dairy bulls. We found extensive CNV of β-defensin genes across all breeds, particularly the Holstein–Friesians. We show that certain genes covary, suggesting that in some cases there are multiple genes on a single copy number variable genomic segment. However, because of the extensive variability between breeds and individuals, refining the breakpoints of these CNV regions will remain a challenge until multiple telomere-to-telomere reference genome assemblies are available across breeds.

β-Defensins are expressed in the epididymis and are likely to be important for fertility and reproduction by maintaining healthy, functioning sperm, either directly by being a component of the glycocalyx, for example, or indirectly by providing an innate immune defence against microbes present in the female reproductive tract [16,59,60]. Defensins that show increased expression in adult testis compared with calf testis are likely to have been upregulated during sexual maturation and therefore be particularly important in the function of the sperm. In addition, some β-defensins show androgen-dependent expression in rats [61]. The defensins we find to be upregulated in bulls include both highly conserved testis-specific defensins with long, potentially glycosylated, C-terminal tails and more recently evolved defensins, shorter, with known antimicrobial and immunomodulatory functions. In a previous study on selected β-defensins, out of the 14 found to be upregulated in our study, *DEFB119* had been found by RT-PCR to be absent in calf testis but highly expressed in adult testis [47]. Taken together, our data suggest defensins with a variety of functional roles are important in fertility and highlight a selected number to be prioritized for further functional analysis.

Sperm maturation occurs in the epididymis, which is divided into caput, corpus and cauda. Region-specific expression of β-defensins has been previously shown in several species [45,48,49,62], including in bull [47]. In this study, we show that almost all β-defensins are expressed in the caput, but show extensive inter-individual variation. A variety of factors may influence expression levels, including age, time and season of sampling, and emphasizes that multiple individuals need to be included in an experiment in order to make secure inferences about tissue- and region-specific expression. Moreover, it is possible that β-defensins expressed in caput epididymis may also play a role in sperm function. For example, in rats, it was determined that *DEFB19* was not detected in the corpus and cauda, but was expressed only in the testis and caput epididymis [45]. Although mature sperm do not carry *DEFB19* mRNA on their own, *DEFB19* signal was observed in the middle part of the sperm. These results suggest that the *DEFB19* signal in sperm is produced in the testis and epididymis and mature sperm indicates that it is protected by sperm [45].

Genetic variation can influence expression levels of a gene, and CNV of β-defensins has been associated with expression levels in humans [26,63,64]. We used the caput expression data to test whether gene copy number accounted for at least part of the expression variation observed between individual bulls. We found that, for four genes, expression level was correlated with copy number, although accounting for a small fraction (less than 14%) of the variation. Interestingly, for two of the genes, the association was negative, suggesting that these effects might be through alterations in regulation/distance from an enhancer or alterations in chromatin structure. The absence of an effect of copy number on expression levels of other genes suggests that extra copies of these genes may be silenced, at least in the epididymis, or the effect on expression is far outweighed by other genetic or environmental factors. In this context, it is noteworthy that a study conducted in the caput epididymis of mice determined that DICER1, along with several β-defensin genes including *DEFB103*, is significantly enriched in their promoters and plays a role in controlling transcription [50]. Such findings highlight that gene regulation in the epididymis will involve other mechanisms in addition to copy number effects.

Taken together, we elucidate the evolutionary history and demonstrate extensive CNV of cattle β-defensins and highlight several genes, most notably *DEFB103*, that are candidates for a functional effect of CNV on fertility. *DEFB103* is associated with the cattle disease mastitis [65], and, in humans, *DEFB103* has a complex role in regulating chemokine and inflammatory cytokine responses to microbial antigenic exposure [52,66]. In particular, *DEFB103* regulates the release of pro-inflammatory cytokines such as *IL6, IL9,* and *TNF* in human myeloid dendritic cells and modulates immune cell responses to specific antigens [67]. Additionally, human *DEFB103* has been shown to suppress pro-inflammatory cytokine production by inhibiting NF-κB signalling and targeting TLR4 pathways in macrophages [68,69]. It has been reported that *DEFB103* could have a therapeutic effect on systemic inflammation associated with periodontal infections by modulating macrophage activation and orientation [70] and determined that it plays a role in the wound repair mechanism by chemotactically attracting macrophages via CCR2 [71]. These functions highlight its role in fine-tuning immune interactions at critical stages influencing the local immune environment [4,52]. The role of *DEFB103* in sperm function and interactions with the female reproductive tract, and thus its potential link to fertility in cattle, remain unclear. Indeed, deletion of the *DEFB103* orthologue *Defb14* has been reported to have no effect on mouse fertility [17]. However, given *DEFB103*’s expression in the caput of the epididymis, its extensive multi-allelic CNV associated with expression levels and its upregulation during sexual maturity, it may participate in reproductive processes in cattle. Therefore, functional characterization of *DEFB103* and the encoded BBD103 protein in reproduction and fertility is a priority.

## Data Availability

European Nucleotide Archive (https://www.ebi.ac.uk/ena) accession numbers for DNA sequencing data are given in electronic supplementary material, tables S1, S2, S3 and S6 [72]. Raw gene copy number estimates by ddPCR and WGS are freely available at Leicester Research Archive [73].
